# Radiologists’ Performance at Reduced Recall Rates in Mammography: A Laboratory Study

**DOI:** 10.31557/APJCP.2019.20.2.537

**Published:** 2019

**Authors:** Norhashimah Mohd Norsuddin, Claudia Mello-Thoms, Warren Reed, Sarah Lewis

**Affiliations:** 1 *Medical Image Optimisation and Perception Group (MIOPeG), Discipline of Medical Radiation Sciences, Faculty of Health Sciences, The University of Sydney, Lidcombe NSW, Australia,*; 2 *Diagnostic Imaging and Radiotherapy Programme, Faculty of Health Sciences, The National University of Malaysia (UKM), Kuala Lumpur, Malaysia.*

**Keywords:** Recall rates, sensitivity, specificity, reader’s performance, screening mammography

## Abstract

**Rationale and objectives:**

Target recall rates are often used as a performance indicator in mammography screening programs with the intention of reducing false positive decisions, over diagnosis and anxiety for participants. However, the relationship between target recall rates and cancer detection is unclear, especially when readers are directed to adhere to a predetermined rate. The purpose of this study was to explore the effect of setting different recall rates on radiologist’s performance.

**Materials and Methods:**

Institutional ethics approval was granted and informed consent was obtained from each participating radiologist. Five experienced breast imaging radiologists read a single test set of 200 mammographic cases (20 abnormal and 180 normal). The radiologists were asked to identify each case that they required to be recalled in three different recall conditions; free recall, 15% and 10% and mark the location of any suspicious lesions.

**Results:**

Wide variability in recall rates was observed when reading at free recall, ranging from 18.5% to 34.0%. Readers demonstrated significantly reduced performance when reading at prescribed recall rates, with lower sensitivity (H=12.891, P=0.002), case location sensitivity (H=12.512, P=0.002) and ROC AUC (H=11.601, P=0.003) albeit with an increased specificity (H=12.704, P=0.002). However, no significant changes were evident in lesion location sensitivity (H=1.982, P=0.371) and JAFROC FOM (H=1.820, P=0.403).

**Conclusion:**

In this laboratory study, reducing the number of recalled cases to 10% significantly reduced radiologists’ performance with lower detection sensitivity, although a significant improvement in specificity was observed.

## Introduction

A comparison of international screening programs has shown a wide range of recall rates in clinical practice across different countries, from 1.4% in the Netherland to 15% in the United States (US) for the first mammography screening examination (Elmore et al., 2003). Recalling a large number of women is considered to improve the number of cancers detected, however previous comparative studies have demonstrated that high recall rates do not significantly improve the cancer detection rate (Kemp Jacobsen et al., 2015; Smith-Bindman et al., 2003). Additionally, a higher recall rate may only contribute to a substantial increase in false positive findings which may result in unnecessary assessments, patient anxiety and additional financial costs hampering the success of breast screening programs (Alamo-Junquera et al., 2012; Bond et al., 2013; Sim et al., 2012). 

The positive correlation between false positive results and recall rates (Gur et al., 2004) has prompted many screening programs to impose specific recall targets in order to optimize the trade-off between recall rates and cancer detection. These recall policies are also used to evaluate the performance of breast readers in the respective programs and provide guidelines for best practice. Variation also exists within screening mammography programs, with higher target recall rates for the first or initial screening as compared to subsequent screening. For example, BreastScreen Australia (BSA) suggests that the clinical recall rate should be at 10% for initial screens with a recall rate for subsequent screening at 5% (BreastScreen Australia, 2008; Perry et al., 2008). 

Extensive studies have shown varying results regarding an appropriate recall rate that will give the best trade-off between recall rates and cancer detection rates (Otten et al., 2005; Schell et al., 2007; Yankaskas et al., 2001). A prospective study by Yankaskas et al. (2001) suggested screening practices at recall rates between 4.9% and 5.5% will yield efficiency in cancer detection versus false positive results, whereas subsequent work by Schell et al., (2007) demonstrated maximum sensitivity and minimal false positives occurred at a 10% recall rate for the first screening and at 6.5% for subsequent screening (Schell et al., 2007; Yankaskas et al., 2001). Another study from the Netherlands has indicated that recall rates of more than 4% only contribute to a higher number of false positive decisions, not the number of cancers detected (Otten et al., 2005). 

The purpose of this study was to explore the effect of setting varying recall rates on the performance of breast readers viewing the same test set of images over three separate reading sessions. We have explored this through a methodology that assesses the radiologists’ ability to correctly locate lesions and give a confidence rating based on the decisions. 

## Materials and Methods

Institutional ethical approval was granted and informed consent was obtained from all participants involved in this study. It was performed at the Medical Imaging Optimisation and Perception Group (MIOPeG) laboratory at the Brain Mind Centre (BMC) of the University of Sydney between February 2015 and January 2016.

*Participants*

Five experienced radiologists who specialized in breast imaging with a median of 15 years (range, 9 to 26 years) of experience of interpreting mammograms, reading between 2,000 and 30,000 (median, 8,000) mammograms each year and spent a median of 10 hours a week reading mammography cases volunteered to participate in this study (Table 1). The participating radiologists were given a small gift voucher as an expression of gratitude for their participation on completion of the study. 

*Experimental protocol*

*Cases*

A test set containing 200 de-identified digital mammographic examinations obtained from the BreastScreen NSW (BSNSW) digital imaging library was presented in a randomized order to each reader for three separate reading sessions. Each case comprised two mammographic views, the cranio-caudal (CC) and the medio-lateral oblique (MLO) respectively of each breast. There were 180 cases with normal findings and 20 cases with abnormal findings in the test set; all the abnormal cases contained a single biopsy-proved malignancy. An expert breast reader who involves in training assessment, quality, clinical policies of BreastScreen NSW and also has responsible for clinical management of a screening center then identified the ‘truth’ locations of all malignant cases. The expert did not participate as an observer in this experiment and had access to prior images with biopsy confirmed malignancy results. Normal cases were validated after 2 years normal screening follow up.

*Reading environment*

This longitudinal study was divided into three separate reading sessions. Each session had a different recall rate condition and was separated from the previous reading by a minimum of two months to reduce any memory effects. The total study time was six months for the three reads for each reader. At the first reading session, no numerical percentage recall rate was imposed and readers were tasked with a “free recall” when interpreting the cases; that is, they could recall as many cases as they believed necessary. In the second session, the number of mammographic cases that readers could recall was set at 30 cases (15%), and reduced to 20 cases (10%) for the third reading. 

The laboratory reading environment was designed to be as authentic to the clinical environment as possible, using an identical clinical workstation as used by BreastScreen New South Wales (BSNSW), Australia. All images in the test set were displayed on a pair of five-megapixel (5MP) EIZO Radioforce GS510 medical-grade monitors (Ishikawa, Japan) with ambient lighting maintained at 20 to 40 lux throughout the reading sessions. Prior to study commencement, calibration was performed on the monitor displays to adhere to the Digital Imaging and Communications in Medicine (DICOM) Part 14 Standard (National Electrical Manufacturers Association, 2004).

*Reading Task*

**Figure 1:**
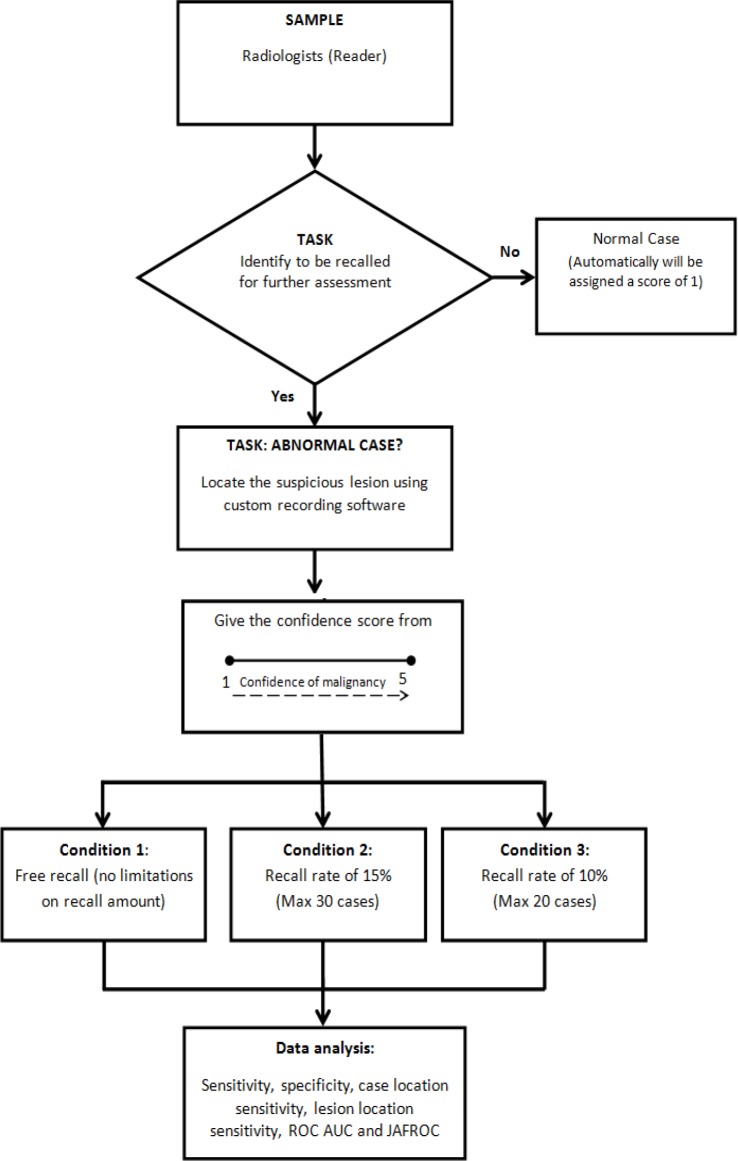
Flowchart Process of Reading Task Conducted in the Study

Figure 1 shows the flowchart process of the study methodology. During the reading sessions, readers identified each mammographic case that they considered malignant in keeping with their free or specified target recall rate. Following that, readers used customized recording software to mark the location of any suspicious lesions from the recalled cases. This software was designed to record all the coordinates of marked lesions for each of the recalled cases on a laptop adjacent to the two 5MP monitors. Readers were not permitted to exceed their target recall rates and the software provided a continuously updated count on the number of cases marked as “recall”. All lesions marked on each recalled case were given a confidence score ranging from 1 to 5, with a greater number indicating a higher confidence of malignancy. A score of 1 or 2 indicated a normal and benign lesion respectively. A 60-pixel acceptance radius surrounding each lesion was considered the acceptable radius as it encompassed the largest lesion present in the test set. A lesion was considered correctly detected when the location was within 60 pixels from the center of the true location of the cancer and it was given a confidence score between 3 to 5. 

**Table 1:** Demographic Details of Participating Radiologists

Reader number	Number of years of experience	Number of mammography cases read per year	Number of hours per week reading mammograms
1	15	30 000	10
2	26	10 000	3
3	15	10 000	10
4	9	6 000	24
5	20	3 500	6
Median	15	8 000	10

**Table 2:** Results for Sensitivity, Lesion Location Sensitivity, Case Location Sensitivity, Specificity, ROC AUC and JAFROC FOM at Free Call, 15% and 10% Conditions

Reader number	Sensitivity			Specificity			Case location sensitivity			Lesion location sensitivity			ROC AUC			JAFROC FOM		
	Free recall	15%	10%	Free recall	15%	10%	Free recall	15%	10%	Free recall	15%	10%	Free recall	15%	10%	Free recall	15%	10%
1	0.8	0.65	0.55	0.83	0.91	0.95	0.8	0.6	0.55	0.78	0.67	0.58	0.84	0.79	0.76	0.83	0.8	0.78
2	0.9	0.65	0Q.45	0.79	0.91	0.93	0.8	0.6	0.45	0.56	0.56	0.47	0.86	0.79	0.7	0.76	0.76	0.71
3	0.9	0.65	0.55	0.72	0.92	0.95	0.8	0.65	0.55	0.81	0.67	0.56	0.84	0.79	0.75	0.8	0.8	0.76
4	0.75	0.7	0.55	0.88	0.92	0.94	0.7	0.65	0.55	0.64	0.64	0.56	0.83	0.82	0.75	0.76	0.79	0.75
5	0.75	0.7	0.55	0.81	0.92	0.95	0.65	0.6	0.55	0.36	0.33	0.5	0.8	0.82	0.76	0.59	0.63	0.73
Median	0.8	0.65	0.55	0.81	0.92	0.95	0.8	0.6	0.55	0.64	0.64	0.56	0.84	0.79	0.75	0.76	0.79	0.75

†, JAFROC FOM, Jack-knife free-response figure of merit; ⱡROC AUC, Receiver operating characteristic area under the curve

**Table 3:** Analysis of Sensitivity, Lesion Location Sensitivity, Case Location Sensitivity, Specificity, ROC AUC and JAFROC FOM

	Kruskal-Wallis Test	Post-hoc test (Mann-Whitney U test)		
		Free recall VS 15%	15% VS 10%	Free recall VS 10%
Sensitivity	0.002*	0.008*	0.006*	0.007*
Specificity	0.002*	0.008*	0.007*	0.008*
Case Location Sensitivity	0.002*	0.013*	0.006*	0.006*
Lesion Location Sensitivity	0.371	0.598	0.243	0.245
ROC AUC	0.003*	0.028	0.009*	0.009*
JAFROC FOM	0.403	0.917	0.251	0.251

*Values shown in bold represent statistically significant difference of Kruskal-Wallis analysis at P value < 0.05 and post hoc Mann-Whitney U test of at P value < 0.017; †JAFROC FOM, Jack-knife free-response figure of merit; ⱡROC AUC, Receiver operating characteristic area under the curve

Readers were not provided with any clinical information associated with the cases including the prevalence of abnormal cases and no prior images were available. The readers had unlimited time and were able to scroll back through the cases if they wished to alter their decision or needed to reduce the number of cases recalled to align with the specific target recall rate condition. Readers were also able to digitally manipulate the images including windowing, zooming and panning as in the actual clinical setting. 

*Data Analysis*

For all reading sessions, reader performance was assessed using sensitivity, specificity, case location sensitivity, lesion location sensitivity, receiver operating characteristic (ROC) area under the curve (AUC) and Jack-knife free-response ROC (JAFROC) figure of merit (FOM). The marked lesions were identified either as positive or negative by comparing selections with the truth table compiled by the expert radiologist. All performance data were analyzed using JAFROC Version 4.2 software and statistical analysis was performed using SPSS software version 22.0. 

A true positive was scored if a lesion was marked within the acceptance radius and received a confidence score between 3 to 5. A false positive was defined for any incorrect localization on normal or benign cases, or if it was outside the 60 pixels range of a lesion in abnormal cases. A true negative outcome was recorded if the case was correctly identified as normal or lesion-free. A false negative was scored when cancer lesions were not marked. The performance metrics used for this study are explained as follows: 

• Sensitivity was defined as the proportion of abnormal cases correctly identified by the reader. 

• Specificity was defined as the proportion of normal cases correctly identified by the reader. 

• Case location sensitivity measures the proportion of positive cases correctly marked, where at least one lesion was correctly identified on the correct location in the case.

• Lesion location sensitivity is the proportion of positive lesions correctly marked;. It was calculated by dividing the number of lesions correctly detected by the total number of positive/abnormal lesions, where the positive lesions detected were on the correct locations for each lesion.

• ROC analysis is a binary paradigm focused on a single rating per case. In this study a TP score was given to a case when the reader correctly identified the correct side of the breast containing cancer, without the need to show the specific location of the lesion.

• JAFROC analysis is a free-response paradigm that allows lesion location information to be included when analysing reader performance. In this study, a TP score was given to a lesion when a reader successfully marked and localized the lesion correctly within the acceptance radius.

The analysis was done in two steps. Firstly, a Kruskal-Willis test was performed across the three reading sessions with statistical significance set at P < 0.05. Secondly, post-hoc analysis using the Mann-Whitney U test was performed to identify which groups were significantly different from each other. For this purpose, Bonferroni adjustment was applied to the alpha values by dividing the alpha level by the number of comparisons made. Results with the revised alpha level, P < 0.017, were deemed to represent significant differences. 

## Results

Table 2 demonstrates the readers’ scores for all performance metrics; sensitivity, specificity, case location sensitivity, lesion location sensitivity, ROC AUC and JAFROC FOM at the conditions of free recall, 15% and 10% recall rate. The median recall rate for all readers when reading at free recall was 25.0% and ranged from 18.5% to 34.0%, which was higher than that recommended by BreastScreen Australia for initial screening (10%). By limiting the number of recalled cases (from free recall to 10% recall rate), readers demonstrated reduced performance, with a decrease in sensitivity (from median 0.80 to 0.55), case location sensitivity (from median 0.80 to 0.55), lesion location sensitivity (from median 0.64 to 0.56), and ROC AUC (from median 0.84 to 0.75). However there was a median increase in specificity from 0.81 to 0.95. 

Significant changes were observed in reduced sensitivity (H=12.891, P=0.002), case location sensitivity (H=12.512, P=0.002) and ROC AUC (H=11.601, P=0.003) along with an increased specificity (H=12.704, P=0.002) across all reading conditions (Table 3). No significant differences were noted for lesion location sensitivity (H=1.982, P=0.371) and JAFROC FOM (H=1.820, P=0.403). Although a significant difference was found in ROC AUC, post-hoc Mann-Whitney U test showed no significant differences in ROC AUC when reading at free recall and 15% (z=-2.200, P=0.028). 

## Discussion

Varied specified recall rates have been introduced by various national and international organizations as a guideline for optimizing the performance of their breast screening programs. However, there is a lack of evidence supporting the actual effect of differing target recall rates upon radiologists’ performance. Our results show that each of the five readers’ sensitivity was highest when operating in the free recall condition as compared to a reduced specified recall rate, with a higher median ROC AUC (0.84) and a JAFROM FOM (0.73). When the readers were tasked with reducing their recalled cases from free recall to specific recall rates (15% and 10%), their performance declined noticeably, with a significant reduction in sensitivity, case location sensitivity and ROC AUC. 

The higher sensitivity at higher specified recall rates observed in our study is in agreement with Gur et al and Schell et al, who suggest that recalling more cases may result in a higher cancer detection rate (Gur et al., 2004; Schell et al., 2007). However, the effectiveness of a breast screening program is not merely dependent on the number of cancers detected but also in reducing the number of unnecessary recall among screened women. It is well documented that false-positive recalls are associated with psychological consequences and economic burden (Bondet al., 2013; Brewer et al., 2007; Brodersen and Siersma, 2013; Castells et al., 2006; Lafata et al., 2004; Maxwell et al., 2013). 

In the current study, readers demonstrated a significant improvement in their specificity as recall rates reduced (P=0.002). By lowering the number of cases allowed to be recalled, readers may have needed to sacrifice some cases that they considered to be abnormal at a higher recall rate, which in turn resulted in fewer false positive decisions. The increased specificity observed here concurs with previous findings by Otten et al and Elmore et al., (2003) where higher recall rates correlated with an increase in false positive findings and lower specificity (Otten et al., 2005). 

Comparing our results directly with previous studies in the literature is complex as we began our reading sessions with the highest recall rate (free recall) and stepped it down to 15% and then 10% which complied with the Australian standard for the first screening recall rate. In the Dutch study by Otten et al., (2005), they reported the effect of increasing recall rates up to 10% on cancer detection, with the best efficiency between cancer detection and specificity seen at recall rates below 5%. Otten et al found that between recall rates of 0.9 and 4.0%, the cancer detection rate increased approximately 17.0% respectively. However, when they modeled a further increase in the recall rate above 5%, the results suggested a relatively small increase in the cancer detection rate (approximately 0.6%), with a higher number of false positive results. 

Similarly, Yankaskas et al., (2001) also reported a positive effect between recall rate and sensitivity at recall rates of 5%. As the recall rates increased to 13.4%, a non-significant increase in sensitivity was observed in this prospective study. In our current work, we were unable to test the effect of lowering the recall rate further to 5% due statistically to the number of cancer cases in the test set of 200. However, the readers in our study demonstrated their best cancer detection at free recall, with a significant decrease in sensitivity at 15% and 10% respectively. In acknowledging a difference in study outcomes, the best explanation may lie in the variation between reading in the clinical environment and our experimental design. Also in contrast to our study in the research by Otten et al., (2005) and Yankaskas et al., (2001), the previous clinical information and prior images were available potentially altering reader performance (Carney et al., 2012; Soh et al., 2013). 

Although small reductions were seen in lesion location sensitivity, there was no statistically significant difference for this performance metric at lower recall rates. Conversely, case location sensitivity demonstrated significant differences across all reading conditions. The discrepancy in these results may be explained by the fact that lesion location sensitivity has a stricter criterion in decision making, where readers must correctly identify the location of the lesion for every lesion in cancer cases. In contrast, the criterion applied for case location sensitivity was for readers to correctly identify where at least one lesion was marked in each case. It also interesting to note that, at an individual level, reader 5 demonstrated a substantial difference in lesion location sensitivity values (below 0.40) as compared to the other four readers, regardless of the recall rate conditions, without a significant change in the case location sensitivity. In this example, we see the clinical reporting behaviour of readers, whereby this reader noted they are more likely to mark only one mammographic view for a case that required further assessment, rather than lesion-specific marking as per our instructions. The reader may have considered the task done if located on just one image as this is still a recall in clinical practice. To our knowledge, no commentary on the effect of recall rates on readers’ performance has been reported using JAFROC analysis from the prior studies. With greater statistical power over traditional methodologies reporting observer’s performance, such as ROC (Chakraborty, 2005, 2006), our data yielded no significant differences in JAFROC FOM as observed across all reading sessions. Quite simply, in the true positive cases that radiologists did recall, they were able to identify the lesion location with good precision.

Although a 10% recall rate in our final specified target recall rate is the same as Australian clinical practice for the first mammographic screening, often readers are not held to this in a strict sense. All five readers in our study demonstrated a higher individual recall rate (free recall) ranging from between 18.5% and 34.0% when no limitation was imposed. This result may be because the readers were aware that the study was conducted in laboratory conditions and their decisions would not influence patient care and no patients would actually be recalled. The readers may also have been expecting a higher number of abnormalities, or an enriched test set, in a laboratory study (Gur et al., 2007; Soh et al., 2013). Additionally, the removal of clinical history and prior images in the test set may have affected their behavior when interpreting the mammograms as they usually have access to this additional information in clinical practice and more likely to recall in these circumstances to be on the safe side.

Our study was designed to resemble clinical mammography practice, hence there was a requirement to use a reasonably high number of cases (n=200) and images (n=800). Therefore fatigue may be present for some readers and this may have contributed to some loss of attention, particularly for difficult to detect lesions (Ciatto et al., 2005). Our sample consisted of highly experienced radiologists (median of 15 years) with more than 3000 mammographic cases reading per year, which may have contributed to the similarity in their performance. A larger study with a diversity of reader experience and case load would allow clarification of the importance of experience when adhering to specific recall rates. It would be interesting to explore how readers with a range of experience adhere to different recall rates and also how varying recall rates would affect the readers’ performance, and may yield some important relationships between lesion location sensitivity and experience. Nevertheless current data from our preliminary work has demonstrated important empirical evidence on how limiting the number of recalled cases in screening can impact on the behavior of readers. A unique aspect of this study is that recall rates were controlled by treating predetermined recall rates as the primary indicator; a confounder known to increase variability in past studies (Otten et al., 2005; Schell et al., 2007; Yankaskas et al., 2001). 

In conclusion, our data suggests that specific target recall rates caused a significant reduction in readers’ sensitivity with an associated increase in specificity. However, no differences in readers’ JAFROC scores were demonstrated, indicating a level of consistency in readers identifying and locating lesions within this test set. Reducing recall rates by enforcing a specific target recall rate may result in a corresponding reduction in cancer detection and may impact on the behavior of readers in their recall-decision strategies for abnormal cases. Further work on how specific lesion features or characteristics were reflected in the readers’ recall decision is required to understand further the nature of improved the balance between true and false positive decisions.
